# Modeling ovarian cancer chemoresistance: establishment of murine ID8 sublines and elucidation of adaptive mechanisms

**DOI:** 10.3389/fonc.2026.1898390

**Published:** 2026-09-11

**Authors:** Shanzhou Xie, Shiqiang Luo, Aozhen Bi, Lizhou Shi, Ying Zhang, Fuqiang Yin, Xia Liu

**Affiliations:** 1 Key Laboratory of Longevity and Aging-related Diseases of Chinese Ministry of Education, Guangxi Medical University, Nanning, Guangxi, China; 2 Institute of Neuroscience and Guangxi Key Laboratory of Brain Science, School of Basic Medical Sciences, Guangxi Medical University, Nanning, Guangxi, China; 3 Department of Clinical Laboratory, The Reproductive Hospital of Guangxi Zhuang Autonomous Region, Nanning, Guangxi, China; 4 Life Sciences Institute, Guangxi Medical University, Nanning, Guangxi, China; 5 Key Laboratory of High-Incidence-Tumor Prevention and Treatment (Guangxi Medical University), Ministry of Education, Nanning, Guangxi, China; 6 Guangxi Health Commission Key Laboratory of Basic Research on Brain Function and Disease, Guangxi Medical University, Nanning, Guangxi, China; 7 Key Laboratory of Human Development and Disease Research (Guangxi Medical University), Education Department of Guangxi Zhuang Autonomous Region, Guangxi Medical University, Nanning, Guangxi, China

**Keywords:** chemoresistance mechanisms, drug-resistant cell lines, ID8 cells, multidrug resistance, ovarian cancer

## Abstract

**Introduction:**

Acquired resistance to platinum-taxane chemotherapy is a major cause of treatment failure in ovarian cancer (OC). *In vitro* cell models are indispensable tools for investigating chemoresistance mechanisms, and the establishment of new resistant cell models contributes to a more comprehensive understanding of these mechanisms. Currently, syngeneic mouse cell models remain scarce.

**Methods:**

Paclitaxel- and carboplatin-resistant sublines (ID8-T and ID8-C) were established by intermittent drug exposure from parental ID8 cells. Cellular morphology was assessed by Giemsa staining. Cell viability was determined using CCK-8 and colony formation assays. Flow cytometry was employed to analyze cell cycle distribution and apoptosis rate. Intracellular drug concentration was quantified by high-performance liquid chromatography (HPLC). Reactive oxygen species (ROS) levels were detected with a fluorescent probe, and protein expression profiles were analyzed by Western blotting.

**Results:**

Both ID8-T and ID8-C exhibited significant resistance to their inducing agents, with resistance indices of 4.54 and 2.21, respectively, and demonstrated cross-resistance phenotype. They showed enhanced clonogenic survival and attenuated drug-induced cell cycle arrest and apoptosis. Mechanistically, resistant cells exhibited reduced intracellular drug accumulation, maintained lower ROS levels, and displayed altered expression of key regulatory proteins. These included sustained pro-survival signals (MCM2, STAT3, 4E-BP1), impaired apoptotic execution (reduced cleaved caspase-3/PARP/LaminA/C), upregulation of the drug efflux pump ABCB1, and dysregulated mitochondrial fission machinery (downregulated MFF).

**Conclusion:**

We have established and characterized two novel murine OC chemoresistance models. The resistant phenotypes of ID8-T and ID8-C sublines are associated with a multifaceted adaptive signature involving enhanced drug efflux, evasion of cell cycle arrest and apoptosis, reduced ROS levels, and altered expression of proteins related to mitochondrial fission. These well-characterized syngeneic models provide valuable tools for future mechanistic and therapeutic studies aimed at overcoming chemotherapy resistance in OC.

## 1 Introduction

Ovarian cancer (OC) remains the most lethal gynecological malignancy worldwide, with approximately 324,398 new cases diagnosed and 206,839 deaths reported annually (1). The standard first-line treatment for OC involves cytoreductive surgery combined with platinum- and paclitaxel-based chemotherapy (2). Although initial response rates are high, over 70% of patients eventually experience recurrence and develop chemotherapy resistance, leading to treatment failure and poor prognosis (3). Therefore, elucidating the molecular mechanisms underlying chemotherapy resistance, particularly to first-line agents such as paclitaxel and carboplatin, is an urgent clinical need.

Stable *in vitro* drug-resistant cell models are indispensable tools for studying resistance mechanisms. Resistant sublines can be established from parental cells through long-term, incremental exposure to drugs, enabling research into related resistance pathways. For example, studies using the platinum-resistant OC model A2780/DDP have revealed that YTHDF1-mediated m^6^A modification promotes cisplatin resistance via the FZD7/Wnt/β-catenin pathway (4); Similarly, research employing the paclitaxel-resistant cell line SKOV3-PTX has demonstrated that STAT1 can reverse resistance by promoting apoptosis through the FAS/CASP8 signaling pathway (5). These studies highlight the importance of *in vitro* drug-resistant cell models. However, a major limitation of these human OC-resistant cell lines is their inability to be directly transplanted into immunocompetent animals to establish syngeneic models, which restricts their application in simulating the complete tumor microenvironment and in conducting *in vivo* efficacy and mechanistic validation studies.

In 2000, Katherine F. Roby et al. first established the mouse OC cell line ID8 (6), a syngeneic mouse OC model derived from C57BL/6 mice. Due to its similarity to high-grade serous OC and its advantage in easily constructing syngeneic transplant tumor models, the ID8 cell line—both in its wild-type and p53-deficient forms—has been widely used in studies of tumorigenesis, metastasis, and treatment response. For instance, Zoe R. C. Marks et al. established mouse models of primary and advanced OC by orthotopic and intraperitoneal injection of ID8 cells into C57BL/6 mice and demonstrated that recombinant Interferon-ϵ treatment significantly suppressed the growth of peritoneal metastases (7). Chin-Jui Wu utilized ID8 cells to develop a mouse epithelial ovarian cancer (EOC) model and found that loss of Lect2 promotes EOC progression by modulating both tumor cells and the tumor microenvironment (8). Gupta et al. showed that ID8 cells undergo phenotype switching depending on the local tissue microenvironment, underscoring the relevance of this syngeneic model for studying HGSOC progression and therapy (9). However, reports on drug resistance studies utilizing this cell line remain scarce, particularly those involving multidimensional characterization of resistance to two frontline agents with distinct mechanisms of action—paclitaxel and carboplatin. The absence of such well-characterized models has limited our understanding of the phenotypic and molecular alterations associated with acquired resistance to these two drugs in a syngeneic setting.

To bridge this gap, we established and multidimensionally characterized two novel ID8-derived resistant sublines—ID8-T (paclitaxel-resistant) and ID8-C (carboplatin-resistant)—through a series of *in vitro* experiments, including assessments of cell morphology, viability, clonogenicity, cell cycle, apoptosis, drug accumulation, reactive oxygen species (ROS) levels, and expression of key signaling proteins. This work provides a multidimensional reference for the phenotypic and molecular features of paclitaxel- and carboplatin-resistant ID8 cells, and serves as a valuable experimental resource for future studies investigating chemoresistance mechanisms in OC.

## 2 Materials and methods

### 2.1 Establishment of drug-resistant ID8 cell lines

The parental mouse ovarian cancer ID8 (Trp53 wild-type) cell line used in this study was maintained in our laboratory and was authenticated by Short Tandem Repeat (STR) profiling. Paclitaxel-resistant (ID8-T) and carboplatin-resistant (ID8-C) sublines were established from the parental ID8 cell line using an intermittent induction method involving repeated cycles of drug exposure and recovery. Over a period of 8 months, cells were exposed to progressively higher concentrations of paclitaxel (ranging from 5 to 45 nM) or carboplatin (ranging from 5 to 50 μM). For each cycle, cells were treated with the drug for 24 h, then cultured in drug-free medium until cells recovered. This cycle was repeated with stepwise increasing concentrations until stable resistant cell lines were obtained. To exclude residual drug interference, the established ID8-T and ID8-C cells were passaged in drug-free medium for at least three passages before cryopreservation. Similarly, following thawing, cells were cultured in drug-free medium for at least three additional passages before being used in any experiments. All cell lines were routinely tested and confirmed negative for mycoplasma contamination. The established ID8-T and ID8-C cells were cryopreserved and maintained in our laboratory for subsequent experiments.

### 2.2 Cell culture

The ID8, ID8-T and ID8-C cells were maintained in Dulbecco’s Modified Eagle Medium (DMEM) (319-006-CL, Wisent, Nanjing, China) supplemented with fetal bovine serum (FBS) (086-150, Wisent, Nanjing, China) penicillin-streptomycin (P1400, Solarbio, Beijing, China) recombinant human insulin (PB180432, Procell, Wuhan, China), sodium selenite (S5261, Sigma-Aldrich, Beijing, China), and human apotransferrin (T1147, Sigma-Aldrich, Beijing, China) and incubated in a humidified cell incubator at 37 °C with 5% CO_2_.

### 2.3 Giemsa staining

Cells were seeded in 12-well plates at a density of 5 × 10³ cells/mL. ID8 and ID8-T cells were treated with 15.625 nmol/L paclitaxel, while ID8 and ID8-C cells were exposed to 62.5 μmol/L carboplatin. After 72 hours of drug treatment, cells were fixed with 4% paraformaldehyde for 30 minutes at room temperature. Subsequently, cells were stained with 1 mL of Giemsa working solution (prepared by mixing 1 mL of Giemsa stock solution with 9 mL of Giemsa dilution buffer) (G1010, Solarbio, Beijing, China) for 20 minutes. Following staining, the dye solution was gently removed by rinsing with distilled water, and the plates were air-dried. Cell morphology was then observed and recorded using an Olympus IX71 inverted fluorescence microscope (Olympus Corporation, Tokyo, Japan).

### 2.4 Cell viability assay

Cells were seeded in 96-well plates at a density of 600 cells per well and allowed to adhere for 16 hours. Subsequently, ID8 and ID8-T cells were exposed to a concentration gradient of paclitaxel (1.953125, 3.90625, 7.8125, 15.625, 31.25, 62.5, and 125 nmol/L), while ID8 and ID8-C cells were treated with increasing concentrations of carboplatin (3.90625, 7.8125, 15.625, 31.25, 62.5, 125 and 250 μmol/L). After 72 hours of drug exposure, cell viability was assessed using a CCK-8 kit (CA1210, Solarbio, Beijing, China). Briefly, the culture medium was aspirated and replaced with 110 μL of serum-free DMEM containing 10 μL of CCK-8 reagent. The plates were then incubated at 37 °C in the dark for 30 min, and the absorbance was measured at 490 nm using a microplate reader. The relative cell viability and drug resistance index (RI) was calculated using the following equation: Relative Viability (%) = (treated group/control group) x 100%. RI = IC_50_ of resistant cell subline/IC_50_ of the parental ID8 cells.

### 2.5 Cross-resistance assay

The cell seeding density, drug exposure concentrations and duration, CCK-8 assay procedure, the calculations of the IC_50_ and RI were performed as described in Section 2.3. For cross-resistance assessment, the drug treatments were modified as follows: ID8 and ID8-C cells were exposed to paclitaxel, while ID8 and ID8-T cells were exposed to carboplatin.

### 2.6 Colony formation assay

Cells were plated in 6-well plates at 150 cells/well. After 16 hours of incubation, ID8 and ID8-T cells were exposed to paclitaxel at concentrations of 1.953125, 3.90625, 7.8125, and 15.625 nmol/L, while ID8 and ID8-C cells were treated with carboplatin at 7.8125, 15.625, 31.25, and 62.5 μmol/L. The drug exposure lasted for 6 days, during which the medium containing drugs was refreshed every 48 hours. After treatment, cells were washed with Dulbecco’s phosphate-buffered saline (D-PBS), fixed with 4% paraformaldehyde for 30 min, and stained with 0.1% crystal violet (G1063, Solarbio, Beijing, China) for 20 min. After staining, digital images of the plates were acquired by scanning, and the area of cell colonies in each well was analyzed using ImageJ software. The relative colony formation rate was calculated as: (colony area of drug-treated group/mean colony area of vehicle control group) × 100%.

### 2.7 Cell cycle analysis

Cells were seeded in 6-cm culture dishes at a density of 1 × 10^4^ cells/mL. After 16 hours of incubation, ID8 and ID8-T cells were treated with paclitaxel at concentrations of 3.90625, 7.8125, and 15.625 nmol/L, while ID8 and ID8-C cells were treated with carboplatin at 15.625, 31.25, and 62.5 μmol/L. Following 72 hours of drug exposure, cells were harvested by trypsinization, washed twice with ice-cold D-PBS, and centrifuged to remove the supernatant. The cell pellet was resuspended in 500 μL of DNA Staining solution, followed by the addition of 5 μL of Permeabilization solution (both from MultiSciences, Hangzhou, China; Cat. No. 70-CCS012). The suspension was incubated in the dark at room temperature for 30 min. Afterwards, the stained cells were filtered through a cell strainer into flow cytometry tubes to obtain a single-cell suspension and analyzed within 1 hour using a CytoFLEX flow cytometer (Beckman Coulter, Brea, CA, USA). After the cell cycle assay, the proportions of cells in each cell cycle phase were analyzed using ModFit LT software.

### 2.8 Cell apoptosis assay

Cells were seeded in 6-cm culture dishes at a density of 1.5 × 10^4^ cells/mL. ID8 and ID8-T cells were treated with paclitaxel at concentrations of 15.625, 31.25, and 62.5 nmol/L, while ID8 and ID8-C cells were exposed to carboplatin at 62.5, 125, and 250 μmol/L for 72 hours. After treatment, cells were harvested by trypsinization, centrifuged, and the supernatant was discarded. The cell pellet was resuspended in 500 μL of ice-cold 1× binding buffer and filtered through a flow cytometry tube to obtain a single-cell suspension. Then, 5 μL of Annexin V-PE reagent was added and mixed gently, followed by the addition of 10 μL of 7-AAD reagent (AP104, MultiSciences, Hangzhou, China). The mixture was incubated at room temperature in the dark for 5 minutes. Samples were analyzed within 1 hour using a CytoFLEX flow cytometer (Beckman Coulter, Brea, CA, USA).

### 2.9 High-performance liquid chromatography

Cells were seeded in 10-cm culture dishes at a density of 6 × 10^4^ cells/mL. ID8 and ID8-T cells were treated with 62.5 nmol/L paclitaxel, while ID8 and ID8-C cells were treated with 125 μmol/L carboplatin. Cells were harvested at 24 and 48 hours post-treatment. Briefly, harvested cells were washed three times with ice-cold D-PBS to remove extracellular drug, resuspended in HPLC-grade methanol (A452, Fisher, Waltham, MA, USA), and lysed by ultrasonication. The lysate was centrifuged, and the supernatant was collected for HPLC analysis. Chromatographic conditions for paclitaxel analysis: Column: Phenomenex C18 reverse-phase column (250 mm × 4.6 mm, 5 μm); Mobile phase: 80% methanol (A) and 20% water (B), isocratic elution at A:B = 80:20 (v/v); Flow rate: 1.0 mL/min; Detection wavelength: 227 nm; Column temperature: 33 °C; Injection volume: 30 μL. Chromatographic conditions for carboplatin analysis: Column: Agilent TC-C18 column (205 mm × 4.6 mm, 5 μm); Mobile phase: 95% methanol (A) and 5% water (B), isocratic elution at A:B = 95:5 (v/v); Flow rate: 1.0 mL/min; Detection wavelength: 229 nm; Column temperature: 25 °C; Injection volume: 30 μL.

### 2.10 Reactive oxygen species detection

Cells were seeded in 24-well plates at a density of 5 × 10³ cells per well. ID8 and ID8-T cells were treated with 15.625 nmol/L paclitaxel, while ID8 and ID8-C cells were exposed to 62.5 μmol/L carboplatin. A positive control group was treated with 40 μg/mL Rosup. After 72 hours of incubation, intracellular ROS levels were measured using a commercial ROS detection kit (S0033, Beyotime, Shanghai, China). Briefly, cells were stained with 500 μL of 10 μmol/L DCFH-DA working solution per well and incubated at 37 °C for 20 min in the dark. Cells were then washed twice with serum-free medium to remove extracellular DCFH-DA, followed by the addition of 500 μL of serum-free basal medium. Green fluorescence intensity was immediately observed and photographed under a fluorescence microscope. The fluorescence intensity was quantified using ImageJ software.

### 2.11 Western blot

Total protein was extracted from cells using RIPA lysis buffer (R0010, Solarbio, Beijing, China) supplemented with protease and phosphatase inhibitor cocktails. Protein concentration was determined using a BCA protein assay kit (P0009, Beyotime, Shanghai, China) according to the manufacturer’s instructions. All samples were diluted to equal concentrations, and 40 μg of protein per sample was loaded and separated by SDS-PAGE at 120 V for 90 min. Proteins were then transferred onto PVDF membranes using a wet transfer system at 300 mA for 120 min at 4 °C. Membranes were blocked with 5% non-fat milk in TBST for 1 h at room temperature and incubated overnight at 4 °C with primary antibodies diluted in TBST. The following primary antibodies were used: Caspase-3 (1:1000, #9662, CST), Cleaved-Caspase-3 (1:1000, #9664, CST), Lamin A/C and Cleaved-Lamin A/C (1:1000, #4777, CST), PARP and Cleaved-PARP (1:1000, #9542, CST), STAT3 (1:1000, #9139, CST), AKT (1:1000, #9272, CST), CDK6 (1:1000, #3136, CST), MFF (1:1000, #84580, CST), DRP1 (1:1000, #8570, CST), ABCB1 (1:1000, #13978, CST), 4E-BP1(1:1000, #9644, CST), MCM2 (1:1000, #3619, CST), and GAPDH (1:5000, #2118, CST). Protein bands were visualized using an enhanced chemiluminescence (ECL) (34580, Thermo Fisher Scientific, Waltham, MA, USA) substrate and imaged with a MiniChemi chemiluminescence 610 imaging system (Sage Creation, Beijing, China). The resulting images were analyzed by densitometry using ImageJ software.

### 2.12 Statistical analysis

Data are presented as the mean ± standard deviation (SD) from at least three independent biological replicates. Statistical analysis was performed using SPSS software (version 26.0). Comparisons among multiple groups were conducted using two-way analysis of variance (ANOVA). Differences in IC_50_ values were analyzed with the Student’s *t* test. A p-value <0.05 was considered statistically significant.

## 3 Results

### 3.1 Morphological characteristics of parental and drug-resistant ID8 cells

**Figure 1:**
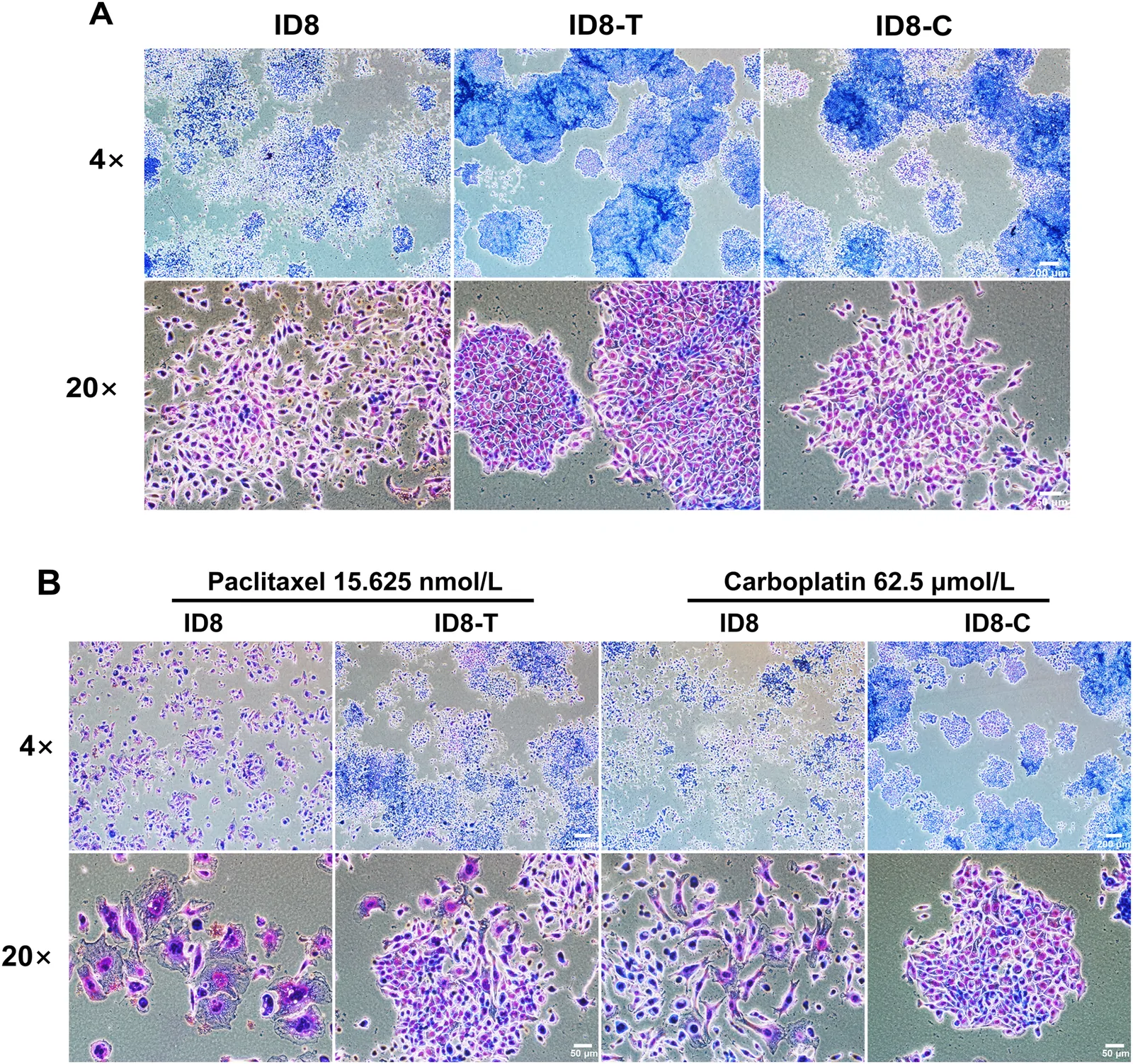
Giemsa staining of ID8, ID8-T, and ID8-C cells. **(A)** Morphology of ID8, ID8-T, and ID8-C cells after 72 h of normal culture. **(B)** Morphological changes after 72 h of drug treatment: ID8 and ID8-T cells were treated with 15.625 nmol/L paclitaxel, while ID8 and ID8-C cells were treated with 62.5 μmol/L carboplatin. Images were acquired at two magnifications: 4× (scale bar = 200 μm) and 20× (scale bar = 50 μm). Representative images from at least three independent experiments are shown.

Cellular morphology was examined by Giemsa staining. In the absence of drug treatment, both ID8-T and ID8-C cells grew in tighter clusters with reduced intercellular spacing compared to the more dispersed parental ID8 cells (**Figure 1A**). Following exposure to paclitaxel (15.625 nmol/L) or carboplatin (62.5 μmol/L) for 72 h, parental ID8 cells exhibited marked cellular enlargement, along with nuclear fragmentation and irregular cell contours. In contrast, the resistant sublines maintained relatively normal cell volume, reached higher confluency, and displayed fewer morphological features of drug-induced damage under the same treatment conditions (**Figure 1B**).

### 3.2 Enhanced drug resistance in ID8-T and ID8-C cells

**Figure 2:**
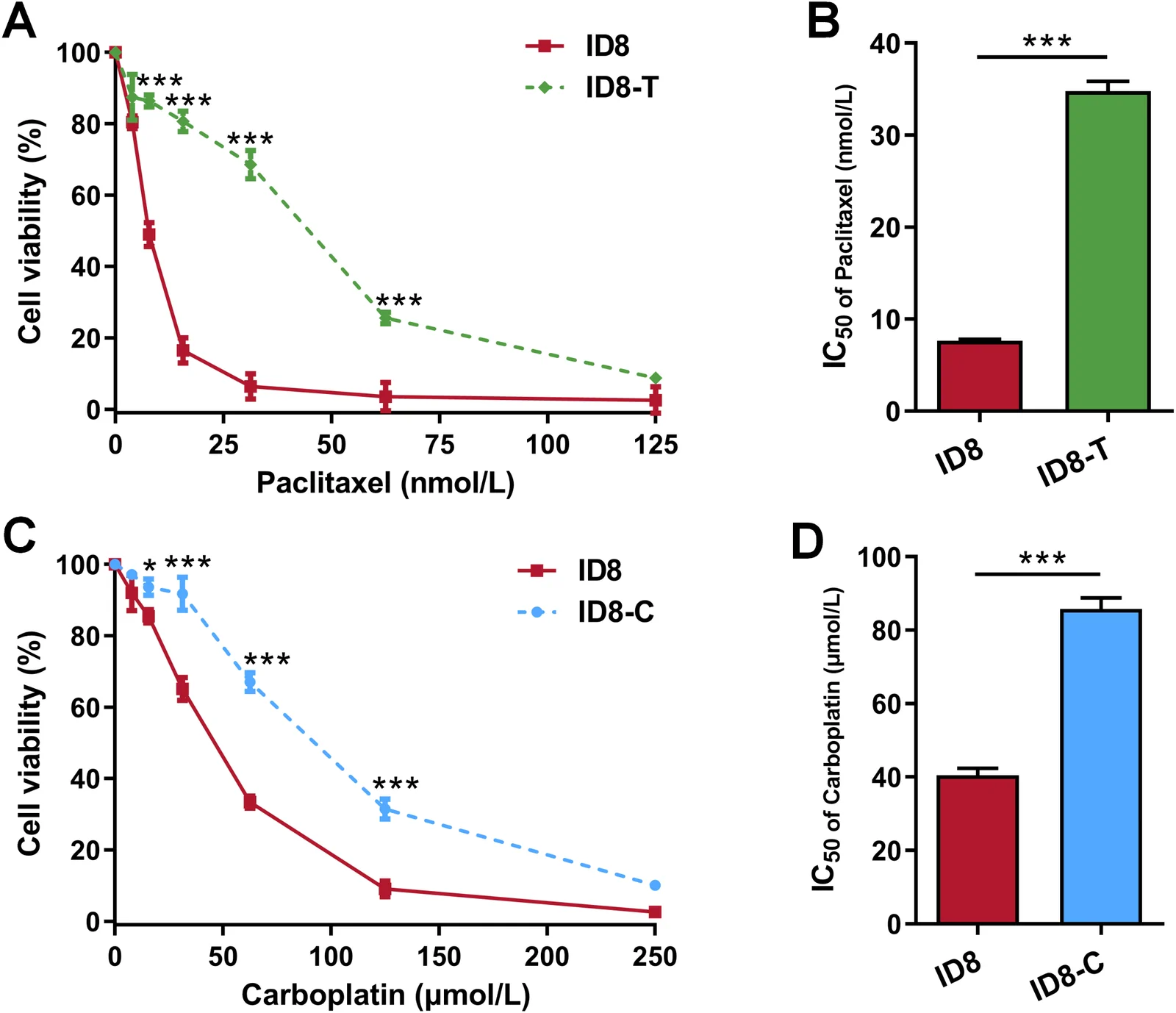
Cell viability of ID8, ID8-T, and ID8-C cells under drug treatment detected by CCK‑8 assay. **(A)** Viability curves of ID8 and ID8-T cells after 72 h of paclitaxel treatment. **(B)** IC50 values of ID8 and ID8-T cells after 72 h of paclitaxel treatment. **(C)** Viability curves of ID8 and ID8-C cells after 72 h of carboplatin treatment. **(D)** IC50 values of ID8 and ID8-C cells after 72 h of carboplatin treatment. Experiments were performed with three biological replicates, and data are presented as mean ± SD. Differences in cell viability between parental and resistant cells at the same drug concentration were analyzed by two‑ay ANOVA. Differences in IC50 values were analyzed by Student’s t‑est. *P < 0.05, **P < 0.01, ***P < 0.001.

Cell viability was measured using the CCK-8 assay after 72 hours of exposure to gradient drug concentrations. As shown in **Figures 2A, B**, ID8-T cells were significantly more resistant to paclitaxel than parental ID8 cells, exhibiting a higher half-maximal inhibitory concentration (IC_50_). The IC_50_ values were 7.65 ± 0.28 nmol/L for ID8 and 34.78 ± 1.83 nmol/L for ID8-T (P <0.001), yielding a resistance index (RI) of 4.54 ± 0.23. Similarly, ID8-C cells showed markedly enhanced resistance to carboplatin, with IC_50_ values of 40.48 ± 3.33 μmol/L for ID8 and 85.78 ± 5.22 μmol/L for ID8-C (P <0.001), corresponding to an RI of 2.21 ± 0.08 (**Figures 2C, D**). These results confirm that both ID8-T and ID8-C sublines possess substantially enhanced resistance to their respective inducing agents.

### 3.3 ID8-T and ID8-C exhibit cross-resistance to alternative chemotherapeutic drugs

**Figure 3:**
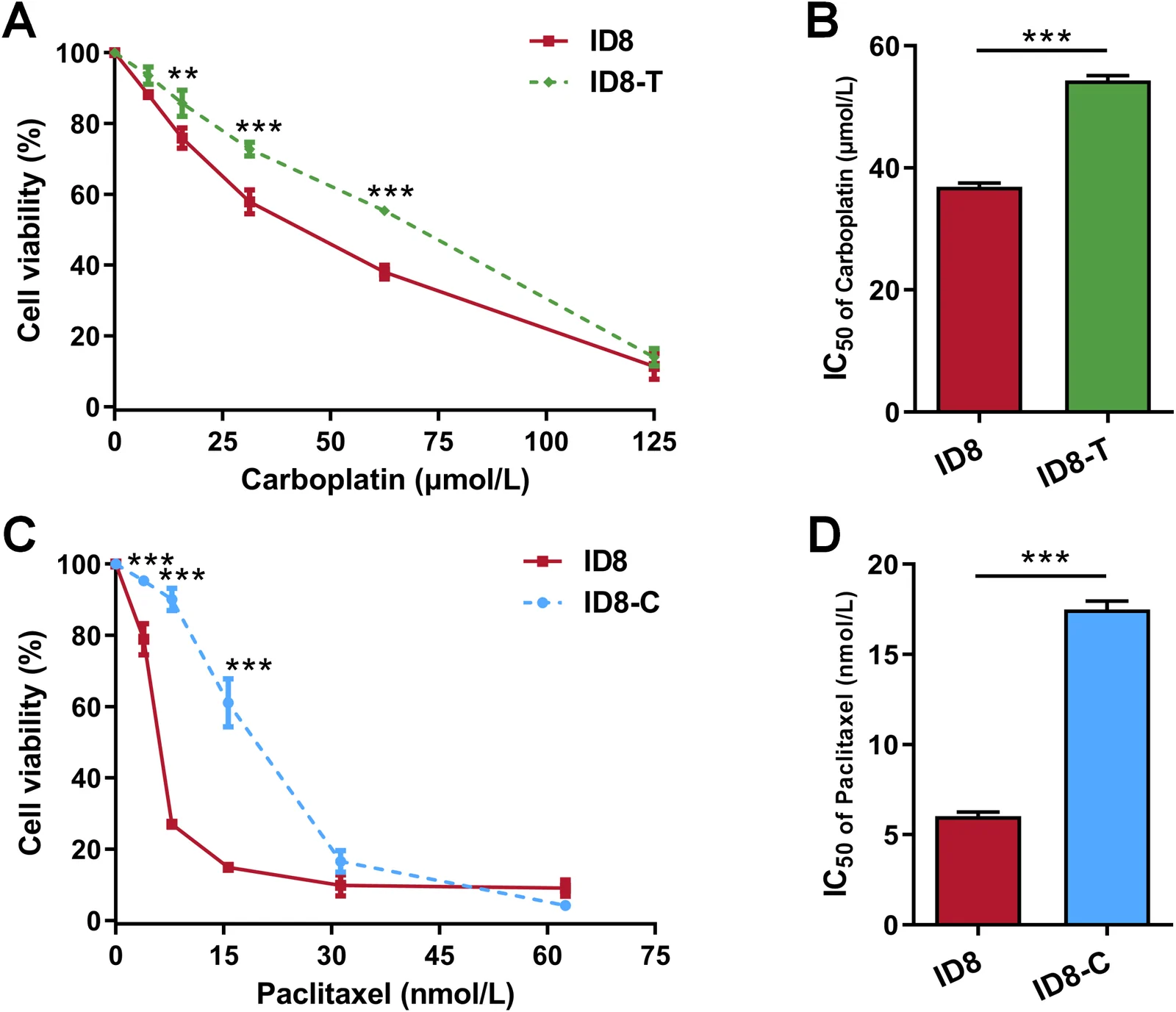
Multidrug resistance of ID8-T and ID8-C cells detected by CCK‑8 assay. **(A)** Viability curves of ID8 and ID8-T cells after 72 h of carboplatin treatment. **(B)** IC50 values of ID8 and ID8-T cells after 72 h of carboplatin treatment. **(C)** Viability curves of ID8 and ID8-C cells after 72 h of paclitaxel treatment. **(D)** IC50 values of ID8 and ID8-C cells after 72 h of paclitaxel treatment. Experiments were performed with three biological replicates, and data are presented as mean ± SD. Differences in cell viability between parental and resistant cells at the same drug concentration were analyzed by two‑ay ANOVA. Differences in IC50 values were analyzed by Student’s t‑est. *P < 0.05, **P < 0.01, ***P < 0.001.

To evaluate cross-resistance, the paclitaxel-resistant subline ID8-T was treated with carboplatin, and the carboplatin-resistant subline ID8-C was treated with paclitaxel for 72 hours, followed by CCK-8 viability assays. As shown in **Figures 3A, B**, ID8-T cells exhibited significantly higher viability and an elevated IC_50_ when exposed to carboplatin compared to parental ID8 cells. The IC_50_ values were 36.91 ± 1.06 μmol/L for ID8 and 54.31 ± 1.39 μmol/L for ID8-T (P <0.001), yielding a resistance index (RI) of 1.41 ± 0.06. Conversely, under paclitaxel treatment, ID8-C cells also showed markedly increased viability and a higher IC_50_ relative to ID8 (**Figures 3C, D**), with IC_50_ values of 6.03 ± 0.41 nmol/L for ID8 and 17.49 ± 0.83 nmol/L for ID8-C (P <0.001), corresponding to an RI of 2.59 ± 0.28. Together, these data demonstrate that both resistant sublines possess cross-resistance, exhibiting reduced sensitivity not only to their primary inducing agent but also to the alternative chemotherapeutic drug.

### 3.4 ID8-T and ID8-C exhibit enhanced colony formation under drug treatment

**Figure 4:**
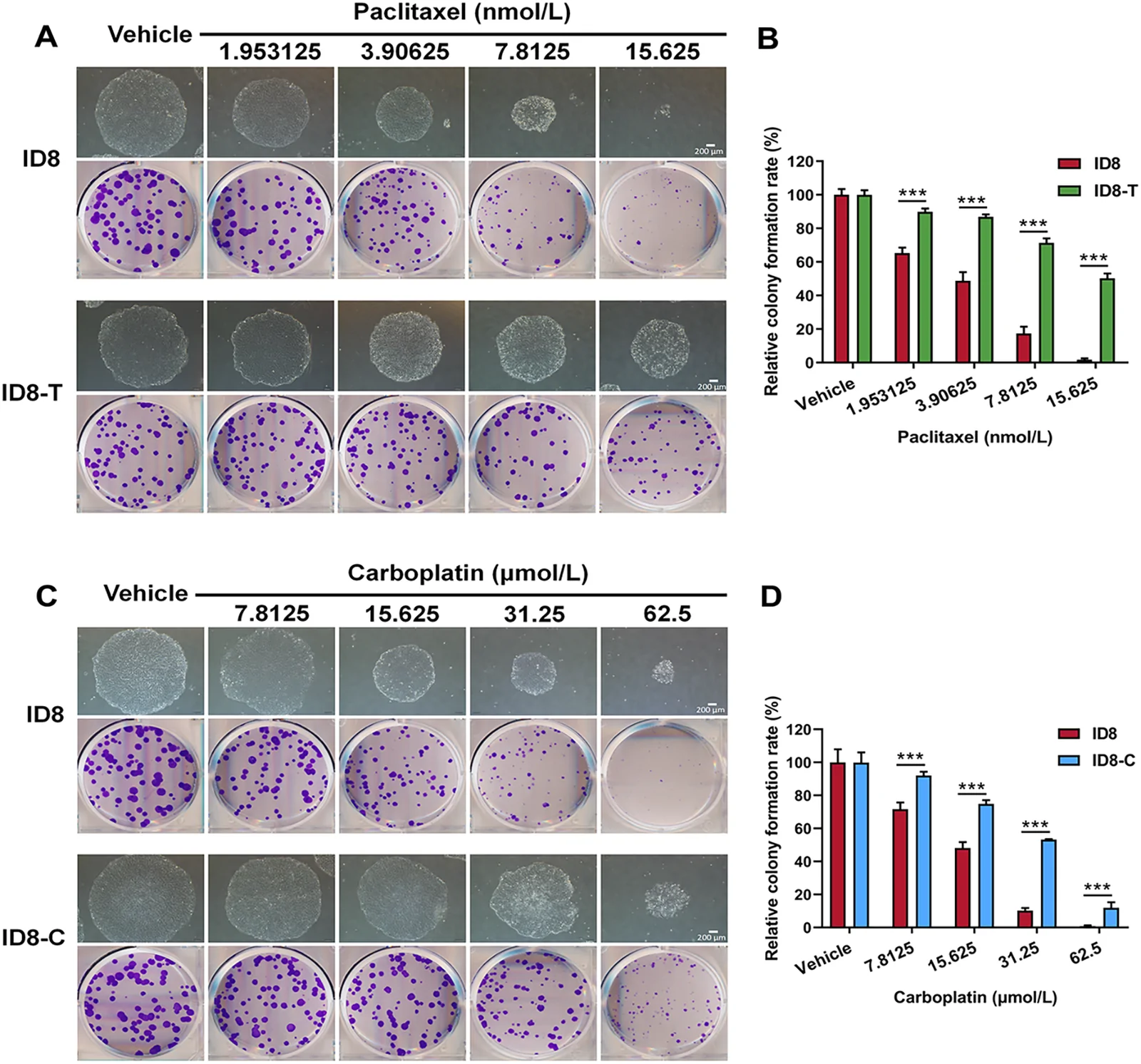
Colony formation ability of ID8, ID8-T, and ID8-C cells under drug treatment detected by plate colony formation assay. **(A)** Representative scanned images of ID8 and ID8-T cells after crystal violet staining, following 6 days of paclitaxel treatment. **(B)** Bar graph of relative colony formation rates of ID8 and ID8-T cells. **(C)** Representative scanned images of ID8 and ID8-C cells after crystal violet staining, following 6 days of carboplatin treatment. **(D)** Bar graph of relative colony formation rates of ID8 and ID8-C cells. Relative colony formation rate is defined as the colony formation rate of cells relative to their respective solvent control groups. Experiments were performed with three biological replicates, and data are presented as mean ± SD. Differences in relative colony formation rates between parental and resistant cells at the same drug concentration were analyzed by two‑ay ANOVA. ***P < 0.001.

Colony formation assays were conducted to assess proliferative capacity and drug sensitivity. Following 6 days of drug exposure, colonies were stained with crystal violet. As shown in **Figure 4A**, ID8-T cells formed significantly more and larger colonies than ID8 cells when treated with 7.8125 and 15.625 nmol/L paclitaxel. Accordingly, the relative colony formation rate of ID8-T was markedly higher than that of ID8 under paclitaxel treatment (P <0.001) (**Figure 4B**). Similarly, in the presence of carboplatin (15.625, 31.25, and 62.5 μmol/L), ID8-C cells produced more and larger colonies compared to ID8 (**Figure 4C**), and exhibited a significantly increased relative colony formation rate (P <0.001) (**Figure 4D**). These results indicate that both ID8-T and ID8-C possess stronger clonogenic survival ability than parental ID8 cells under drug pressure.

### 3.5 ID8-T and ID8-C are less susceptible to drug-induced cell cycle arrest

**Figure 5:**
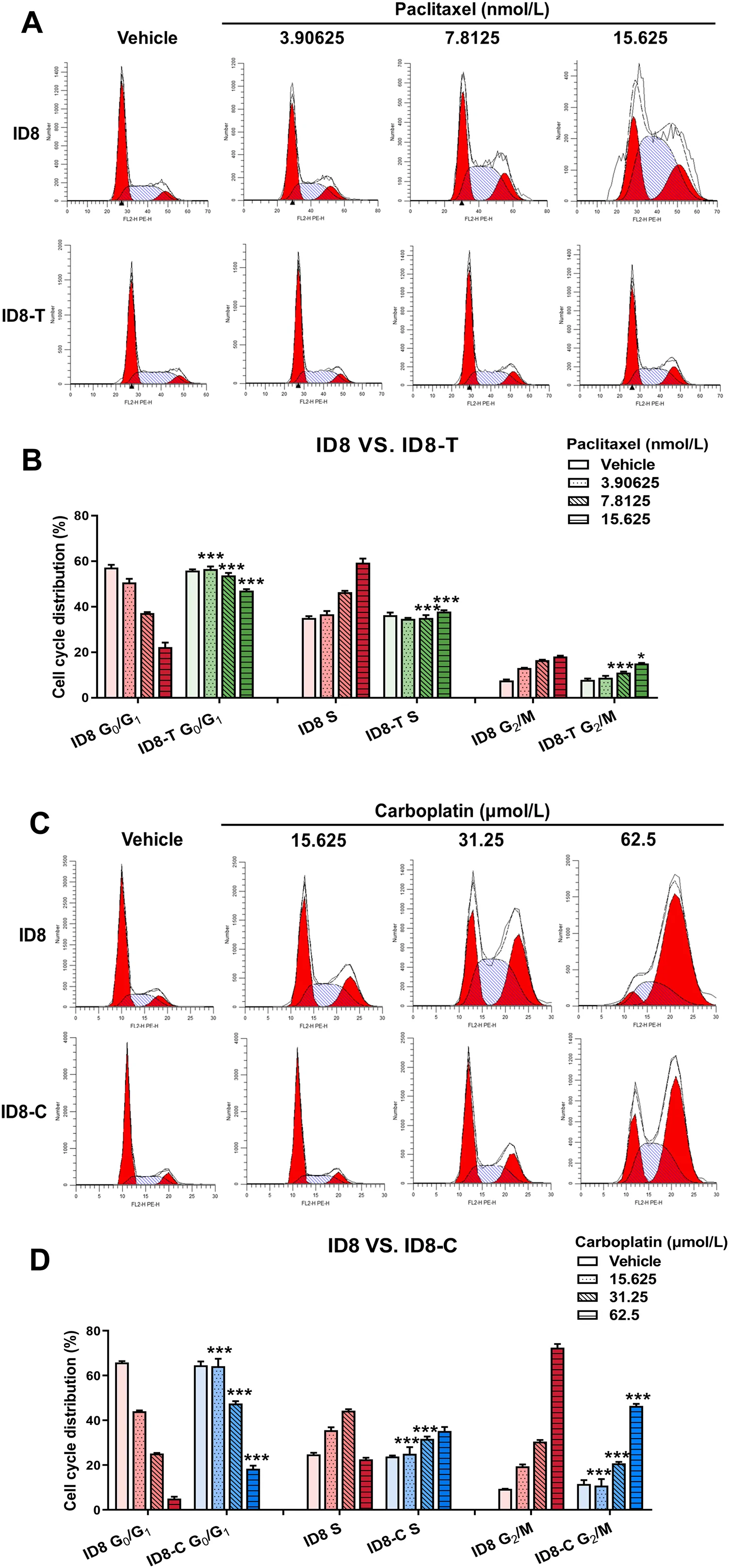
Cell cycle changes of ID8, ID8-T, and ID8-C cells under drug treatment detected by flow cytometry. **(A)** Representative flow cytometry histograms showing cell cycle distribution of ID8 and ID8-T cells after 72 h of paclitaxel treatment. **(B)** Percentage of ID8 and ID8-T cells in G0/G1, S, and G2/M phases. **(C)** Representative flow cytometry histograms showing cell cycle distribution of ID8 and ID8-C cells after 72 h of carboplatin treatment. **(D)** Percentage of ID8 and ID8-C cells in G0/G1, S, and G2/M phases. Experiments were performed with three biological replicates, and data are presented as mean ± SD. Differences in the percentage of cells in G0/G1, S, and G2/M phases between parental and resistant cells under the same drug concentration were analyzed by two‑ay ANOVA. *P < 0.05, **P < 0.01, ***P < 0.001.

As shown in **Figures 5A, B**, paclitaxel treatment induced a pronounced G2/M arrest in parental ID8 cells, accompanied by a marked reduction in G0/G1 phase and an increase in S phase. At 15.63 nmol/L paclitaxel, ID8-T cells exhibited a significantly higher proportion of cells in G0/G1 phase compared to ID8 cells (47.01% ± 0.72% vs. 22.23% ± 2.01%, P <0.001), while displaying significantly lower fractions in G2/M phase (15.10% ± 0.19% vs. 18.18% ± 0.34%, P = 0.046) and S phase (37.89% ± 0.55% vs. 59.31% ± 1.85%, P <0.001). Under carboplatin treatment, ID8-C cells were significantly less affected than parental ID8 cells at each concentration tested (**Figures 5C, D**). At 62.5 μmol/L carboplatin, ID8-C cells retained a significantly higher G0/G1 population compared to ID8 cells (18.37% ± 1.38% vs. 5.01% ± 0.92%, P <0.001), while exhibiting a significantly lower G2/M fraction (46.43% ± 0.92% vs. 72.43% ± 1.67%, P <0.001). At 31.25 μmol/L, ID8-C cells were least affected by carboplatin-induced S phase arrest compared to ID8 cells (31.68% ± 1.11% vs. 44.31% ± 0.65%, P <0.001). Together, these findings demonstrate that both ID8-T and ID8-C cells are significantly more resistant to drug-induced cell cycle arrest than parental ID8 cells.

### 3.6 ID8-T and ID8-C exhibit reduced susceptibility to drug-induced apoptosis

**Figure 6:**
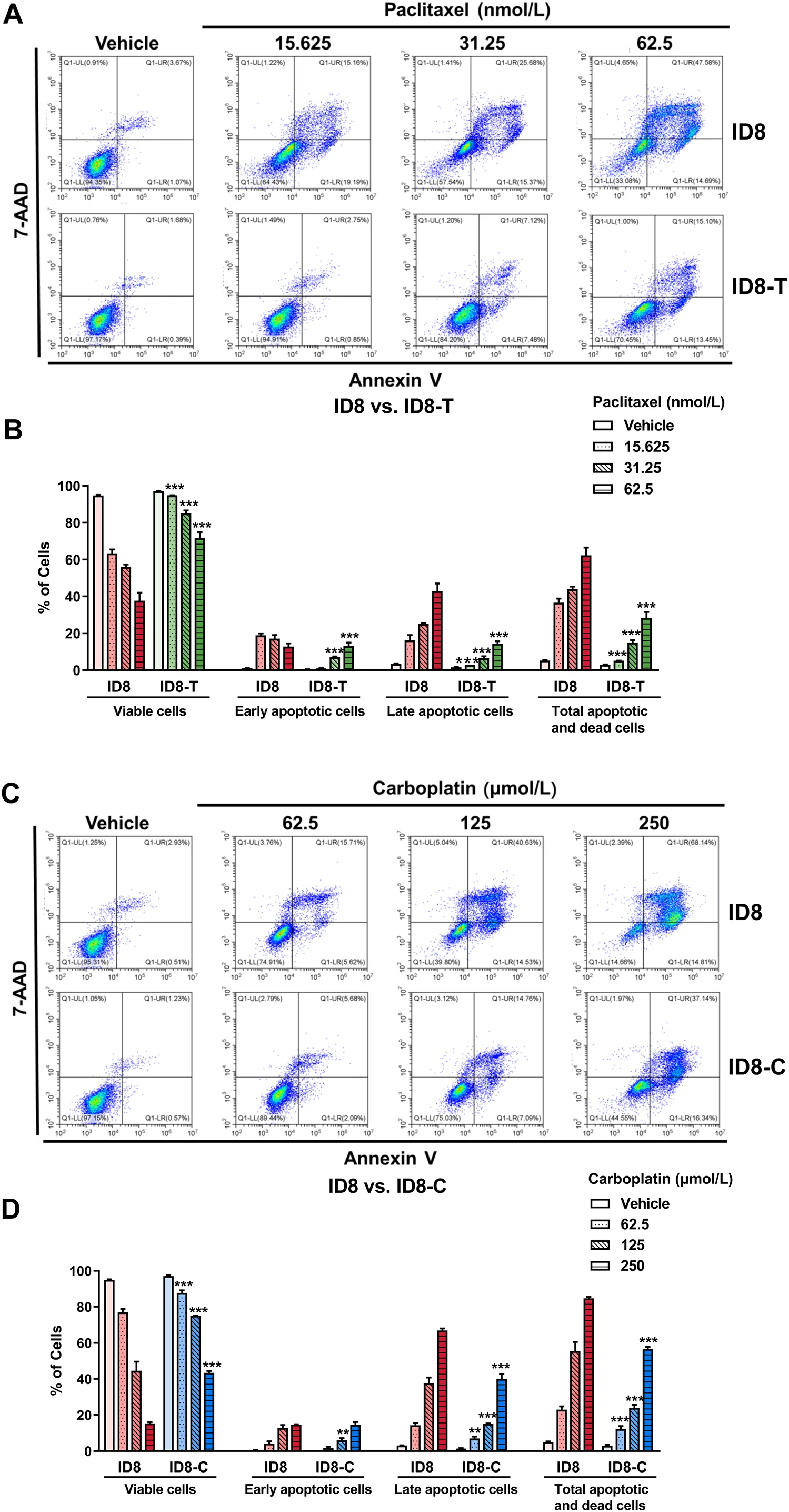
Apoptosis of ID8, ID8-T, and ID8-C cells under drug treatment detected by flow cytometry. **(A)** Representative pseudocolor plots of PE Annexin V/7‑AAD double staining showing apoptosis of ID8 and ID8-T cells after 72 h of paclitaxel treatment. **(B)** Percentages of live cells, early apoptotic cells, late apoptotic cells, and total apoptotic cells in ID8 and ID8-T populations. **(C)** Representative pseudocolor plots of PE Annexin V/7‑AAD double staining showing apoptosis of ID8 and ID8-C cells after 72 h of carboplatin treatment. **(D)** Percentages of live cells, early apoptotic cells, late apoptotic cells, and total apoptotic cells in ID8 and ID8-C populations. In the pseudocolor plots: lower left (LL) quadrant = live cells (Annexin V⁻/7‑AAD⁻); lower right (LR) quadrant = early apoptotic cells (Annexin V⁺/7‑AAD⁻); upper right (UR) quadrant = late apoptotic cells (Annexin V⁺/7‑AAD⁺); upper left (UL) quadrant = necrotic cells or debris (Annexin V⁻/7‑AAD⁺). Total apoptotic cells = sum of LR + UR + UL. Experiments were performed with three biological replicates, and data are presented as mean ± SD. Differences in the percentages of live cells, early apoptotic cells, late apoptotic cells, and total apoptotic cells between parental and resistant cells under the same drug concentration were analyzed by two‑ay ANOVA. *P < 0.05, **P < 0.01, ***P < 0.001.

As shown in **Figures 6A, B**, paclitaxel treatment induced substantial apoptosis in parental ID8 cells, whereas ID8-T cells exhibited marked resistance. At the highest concentration tested (62.5 nmol/L), the total apoptotic rate in ID8 cells reached 62.27% ± 4.36%, compared to only 28.38% ± 3.38% in ID8-T cells (P <0.001), accompanied by a correspondingly higher proportion of viable cells. Both early and late apoptotic fractions were significantly reduced in ID8-T cells relative to ID8 cells. Similar trends were observed at other concentrations (15.625 and 31.25 nmol/L). Consistent with these findings, carboplatin treatment also triggered pronounced apoptosis in ID8 cells, while ID8-C cells were significantly less affected (**Figures 6C, D**). At 125 μmol/L carboplatin, the total apoptotic population in ID8-C cells was markedly lower than that in ID8 cells (23.94% ± 1.75% vs. 55.44% ± 5.09%, P <0.001), with both early and late apoptotic fractions also significantly reduced compared to parental cells. These results demonstrate that both resistant sublines are significantly less susceptible to drug-induced apoptosis compared to parental ID8 cells, indicating that evasion of apoptosis plays a critical role in their acquired drug resistance.

### 3.7 Reduced intracellular drug accumulation in ID8-T and ID8-C cells

**Figure 7:**
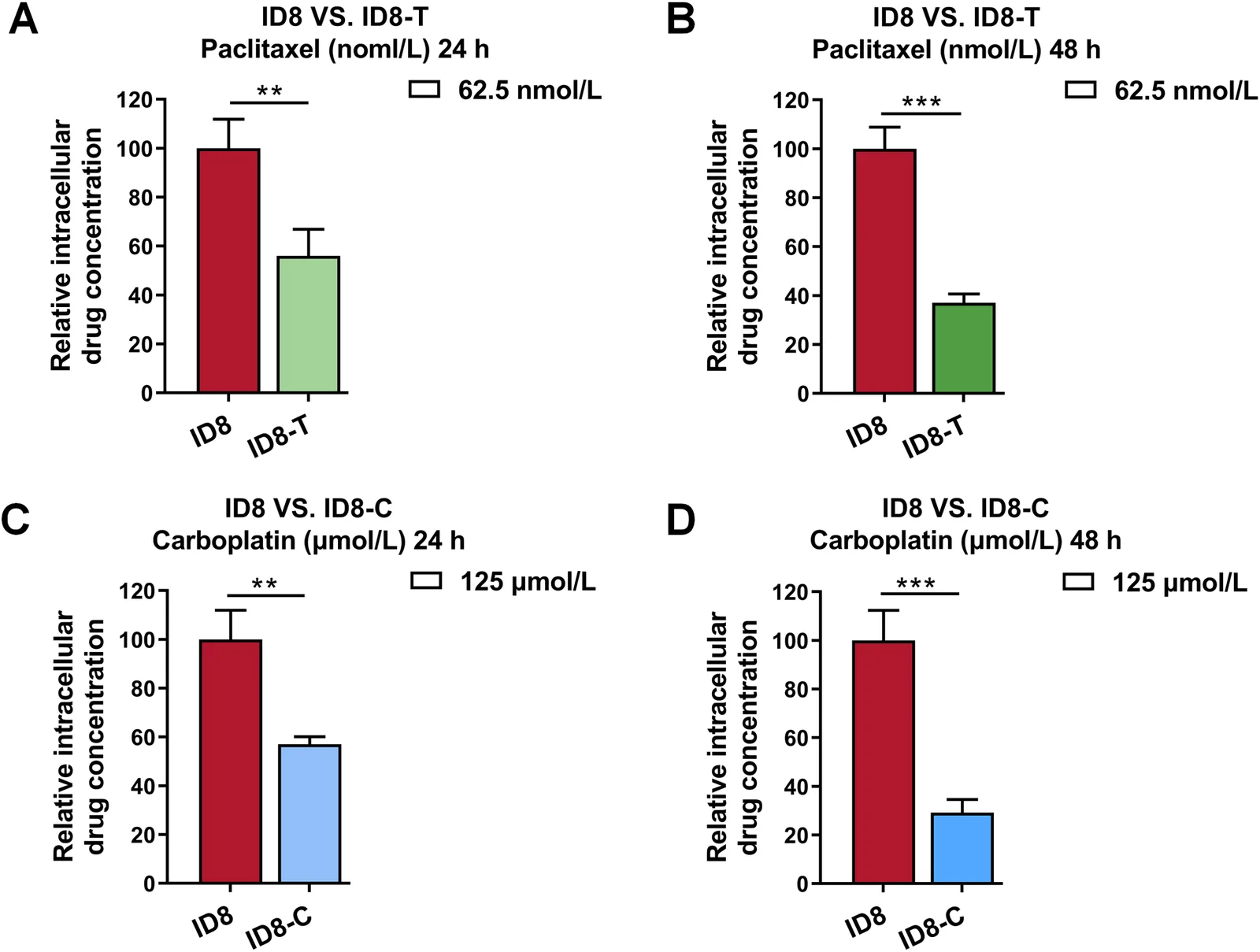
Intracellular drug concentrations in ID8-T and ID8-C cells relative to ID8 cells detected by high‑performance liquid chromatography (HPLC). **(A)** Bar graph showing intracellular paclitaxel concentration in ID8-T cells relative to ID8 cells after 24 h of treatment with 62.5 nmol/L paclitaxel. **(B)** Bar graph after 48 h of treatment with 62.5 nmol/L paclitaxel. **(C)** Bar graph showing intracellular carboplatin concentration in ID8-C cells relative to ID8 cells after 24 h of treatment with 125 μmol/L carboplatin. **(D)** Bar graph after 48 h of treatment with 125 μmol/L carboplatin. The relative intracellular drug concentration is defined as the intracellular drug level in resistant cells (ID8‑ or ID8‑) normalized to that in parental ID8 cells. Experiments were performed with three biological replicates, and data are presented as mean ± SD. Differences in relative intracellular drug concentrations between resistant and parental cells were analyzed by two‑ay ANOVA. *P < 0.05, **P < 0.01, ***P < 0.001.

Enhanced drug efflux is a key mechanism of chemoresistance. To evaluate this, intracellular concentrations of paclitaxel and carboplatin were measured by high-performance liquid chromatography (HPLC) following drug exposure, with values normalized to parental ID8 cells (set as 100%). As shown in **Figures 7A, B**, after treatment with 62.5 nmol/L paclitaxel for 24 h and 48 h, the relative intracellular paclitaxel concentrations in ID8-T cells were 55.98% ± 10.73% (P = 0.009) and 37.11% ± 3.54% (P <0.001), respectively. Similarly, following exposure to 125 μmol/L carboplatin for 24 h and 48 h, ID8-C cells exhibited relative intracellular carboplatin concentrations of 57.01% ± 3.09% (P = 0.019) and 29.23% ± 5.36% (P <0.001), respectively (**Figures 7C, D**). These results demonstrate that both resistant sublines have significantly reduced intracellular accumulation of their respective chemotherapeutic agents relative to parental ID8 cells, supporting enhanced drug efflux as a contributing mechanism of resistance.

### 3.8 ID8-T and ID8-C maintain lower ROS levels under drug stress

**Figure 8:**
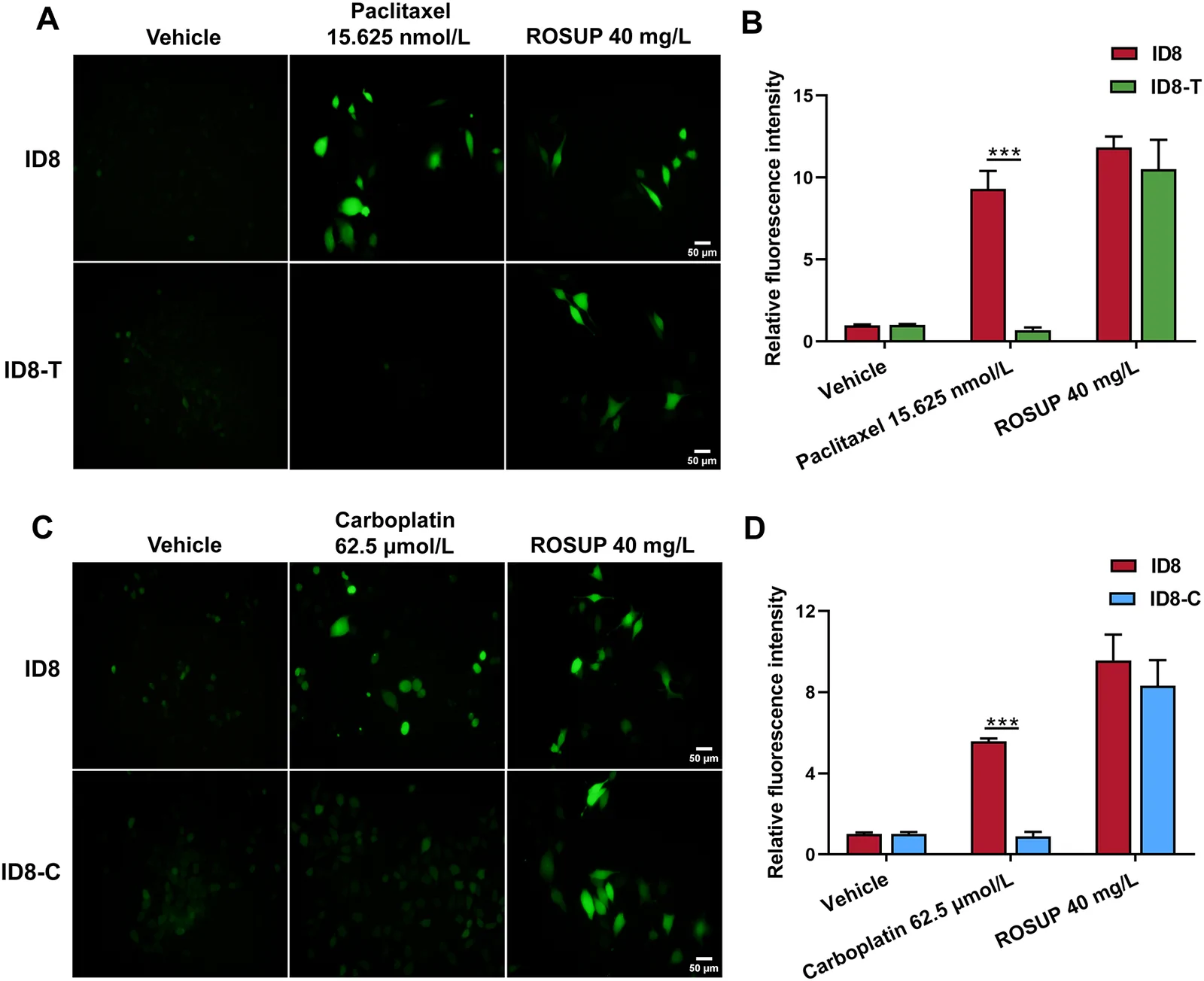
ROS levels in ID8, ID8-T, and ID8-C cells under drug treatment. **(A)** Representative images of DCFH‑DA staining showing ROS levels in ID8 and ID8-T cells after 72 h of treatment with 15.625 nmol/L paclitaxel. Green fluorescence indicates ROS. **(B)** Bar graph of relative fluorescence intensity in ID8 and ID8-T cells. **(C)** Representative images of DCFH‑DA staining showing ROS levels in ID8 and ID8-C cells after 72 h of treatment with 62.5 μmol/L carboplatin. **(D)** Bar graph of relative fluorescence intensity in ID8 and ID8-C cells. Relative fluorescence intensity is defined as the fluorescence intensity of cells normalized to that of their respective vehicle control groups. Experiments were performed with three biological replicates, and data are presented as mean ± SD. Differences in relative fluorescence intensity between parental and resistant cells were analyzed by two‑ay ANOVA. ***P < 0.001.

To assess oxidative stress response, intracellular reactive oxygen species (ROS) levels were measured after 72 hours of drug treatment using a fluorescent probe, with green fluorescence intensity reflecting ROS abundance. As shown in **Figures 8A, B**, paclitaxel (15.63 nmol/L) induced a marked increase in green fluorescence in ID8 cells, whereas ID8-T cells showed no significant change. Quantitative analysis revealed that after paclitaxel treatment, the relative fluorescence intensity in ID8 cells increased from 1.00 ± 0.04 (vehicle) to 9.31 ± 1.10 (P <0.001). Similarly, under carboplatin treatment (62.5 μmol/L), ID8 exhibited a pronounced rise in ROS (from 1.00 ± 0.08 to 5.57 ± 0.15, P <0.001), while ID8-C maintained a relatively stable fluorescence signal (**Figures 8C, D**). In contrast, no significant differences in ROS were observed among ID8, ID8-T, and ID8-C cells under vehicle or after treatment with the ROS inducer ROSUP (**Figure 8**). These results suggest that while paclitaxel and carboplatin elevate ROS in parental ID8 cells, both ID8-T and ID8-C can maintain ROS homeostasis upon drug exposure.

### 3.9 Altered expression profiles of key proteins in resistant sublines revealed by western blot analysis

**Figure 9:**
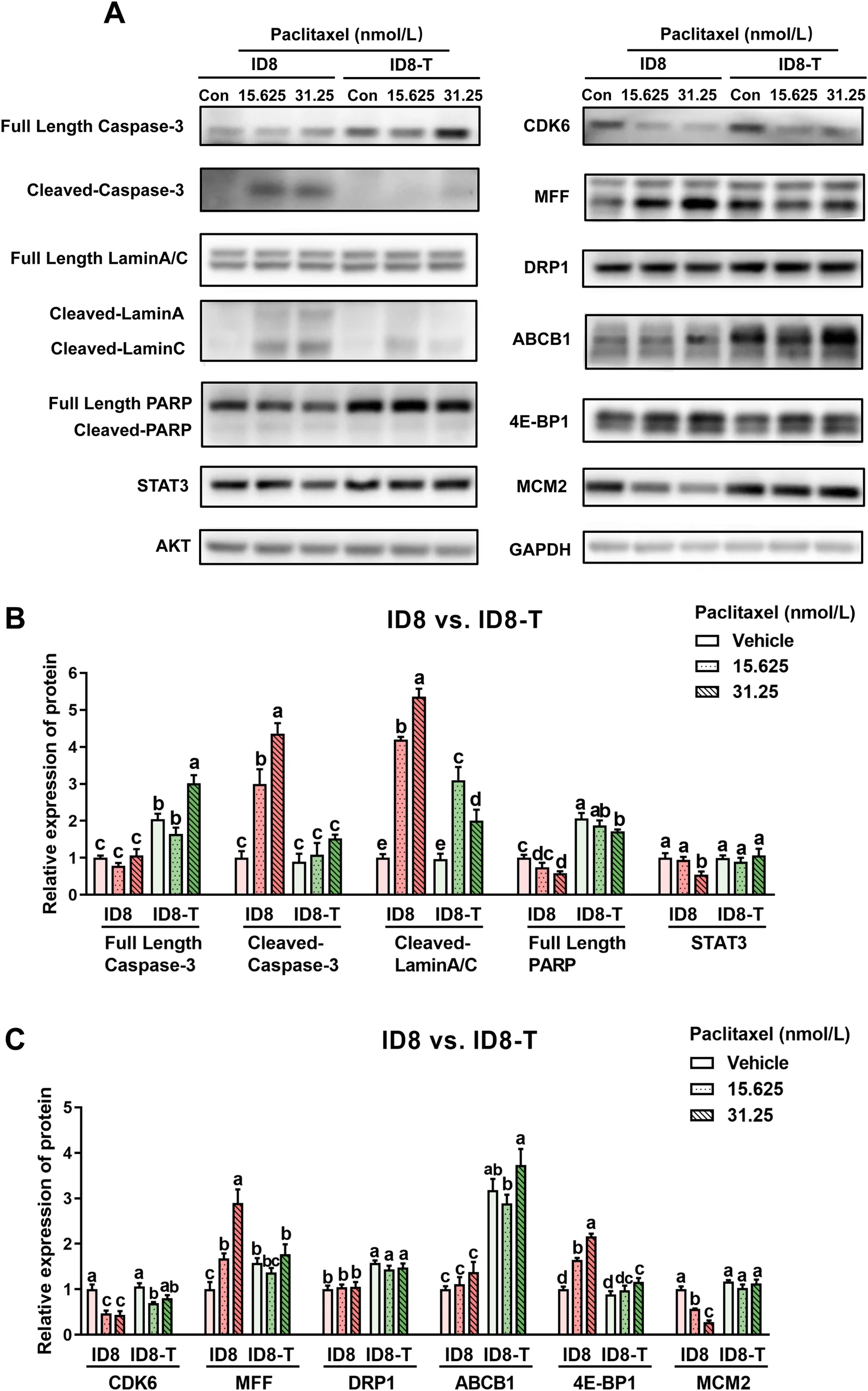
Western blot analysis of proteins related to cell growth, cell cycle, apoptosis, mitochondrial fission, and multidrug resistance in ID8 and ID8-T cells under paclitaxel treatment. **(A)** Representative blots showing protein expression levels of full‑length caspase‑3, cleaved‑caspase‑3, full‑length lamin A/C, cleaved‑lamin A/C, full‑length PARP, cleaved‑PARP, STAT3, AKT, CDK6, MFF, DRP1, ABCB1, 4E‑BP1, and MCM2 in ID8 and ID8‑T cells after 72 h of paclitaxel treatment. GAPDH was used as a loading control. **(B)** Bar graphs of relative expression levels of full‑length caspase‑3, cleaved‑caspase‑3, cleaved‑lamin A/C, full‑length PARP, and STAT3 in ID8 and ID8‑T cells. **(C)** Bar graphs of relative expression levels of CDK6, MFF, DRP1, ABCB1, 4E‑BP1, and MCM2 in ID8 and ID8‑T cells. Relative protein expression levels were calculated by normalizing each treatment group (ID8 treated with 15.625 or 31.25 nmol/L paclitaxel; ID8‑T vehicle or treated with 15.625 or 31.25 nmol/L paclitaxel) to the ID8 vehicle control group. Experiments were performed with three biological replicates. Differences among groups were analyzed by two‑way ANOVA. Different lowercase letters (a, b, c, …) above the bars indicate statistically significant differences between groups (P < 0.05).

**Figure 10:**
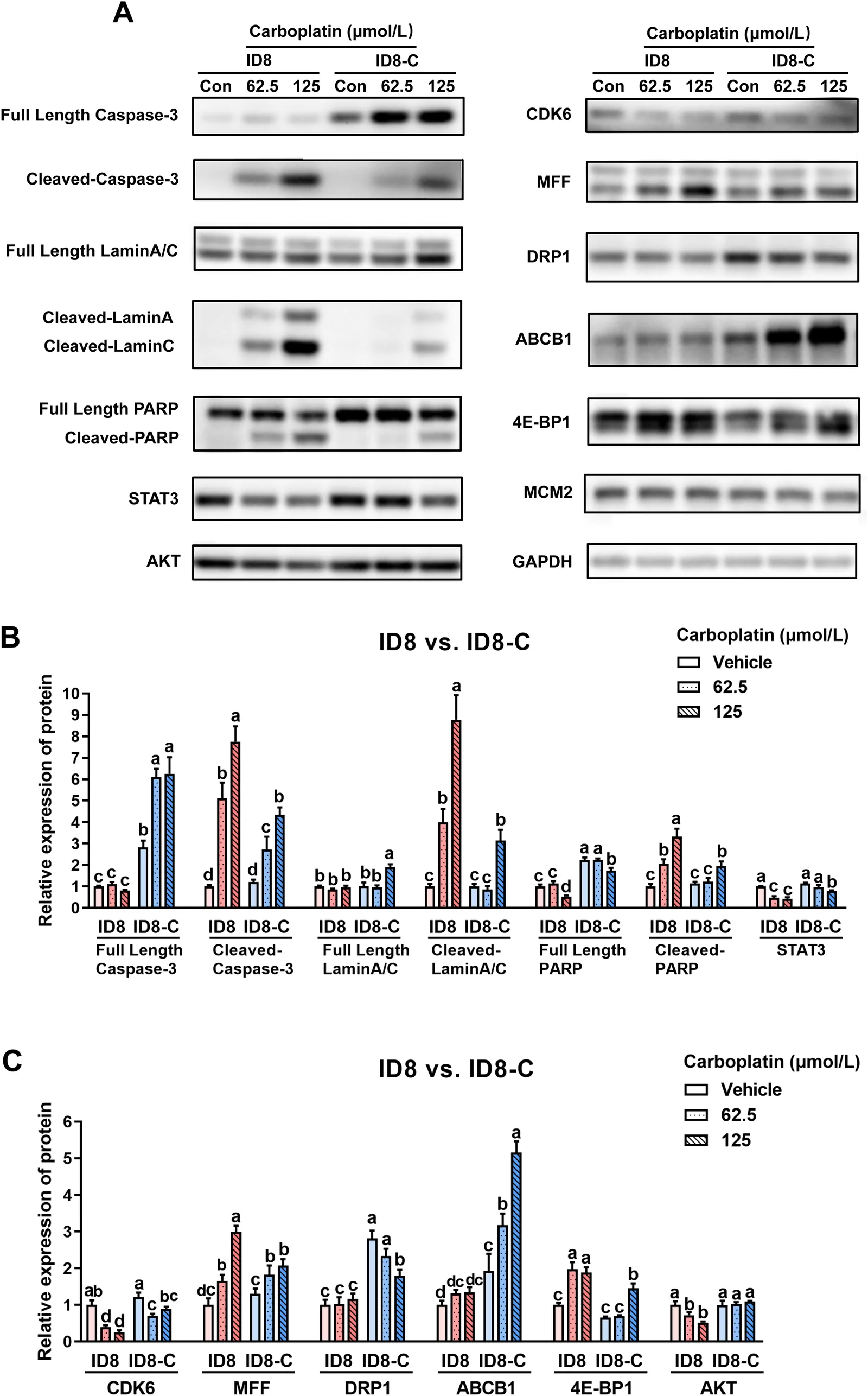
Western blot analysis of proteins related to cell growth, cell cycle, apoptosis, mitochondrial fission, and multidrug resistance in ID8 and ID8‑C cells under carboplatin treatment. **(A)** Representative blots showing protein expression levels of full‑length caspase‑3, cleaved‑caspase‑3, full‑length lamin A/C, cleaved‑lamin A/C, full‑length PARP, cleaved‑PARP, STAT3, Akt, CDK6, MFF, DRP1, ABCB1, 4E‑BP1, and MCM2 in ID8 and ID8‑C cells after 72 h of carboplatin treatment. GAPDH was used as a loading control. **(B)** Bar graphs of relative expression levels of full‑length caspase‑3, cleaved‑caspase‑3, full‑length lamin A/C, cleaved‑lamin A/C, full‑length PARP, cleaved‑PARP, and STAT3 in ID8 and ID8‑C cells. **(C)** Bar graphs of relative expression levels of CDK6, MFF, DRP1, ABCB1, 4E‑BP1, and Akt in ID8 and ID8‑C cells. Relative protein expression levels were calculated by normalizing each treatment group (ID8 treated with 62.5 or 125 μmol/L carboplatin; ID8‑ vehicle or treated with 62.5 or 125 μmol/L carboplatin) to the ID8 vehicle control group. Experiments were performed with three biological replicates. Differences among groups were analyzed by two‑ay ANOVA. Different lowercase letters (a, b, c, …) above the bars indicate statistically significant differences between groups (P < 0.05).

Western blot analysis revealed that, relative to parental ID8 cells, both paclitaxel-resistant ID8-T and carboplatin-resistant ID8-C sublines exhibited pronounced alterations in proteins governing cell growth, cell-cycle progression, apoptosis, mitochondrial fission, and multidrug resistance (**Figures 9**, **10**). In paclitaxel-treated ID8-T cells (**Figure 9**), the growth-related proteins MCM2 and STAT3 were sustained. At 31.25 nmol/L paclitaxel, MCM2 and STAT3 levels were 4-fold (P <0.001) and 1.96-fold (P = 0.002) higher, respectively, than in ID8 cells. The downregulation of CDK6 was attenuated, with its expression 1.82-fold greater than that in ID8 cells (P <0.001). With regard to apoptotic proteins, full-length caspase-3 and PARP were upregulated (2.8-fold, P <0.001 and 1.96-fold, P <0.001), whereas cleaved caspase-3 and cleaved lamin A/C were reduced by 2.8-fold (P < 0.001) and 2.67-fold (P <0.001) compared with ID8 cells at 31.25 nmol/L. Mitochondrial dynamics were altered: MFF upregulation was attenuated by 1.63-fold (P <0.001), and basal DRP1 expression was elevated by 1.40-fold (P <0.001). Of note, the multidrug resistance transporter ABCB1 was markedly upregulated, being 3.18-, 2.60-, and 2.70-fold higher than in ID8 cells at 0, 15.625, and 31.25 nmol/L, respectively (all P <0.001).

Similarly, in carboplatin-treated ID8-C cells (**Figure 10**), STAT3 and CDK6 downregulation was milder, with levels 1.81-fold (P <0.001) and 3.56-fold (P <0.001) higher than in ID8 cells at 125 μmol/L. AKT expression remained largely unaffected by the drug in ID8-C cells, whereas it was downregulated 1.94-fold in ID8 cells (P <0.001). Evaluation of apoptotic markers showed that full-length caspase-3 was elevated (2.82-, 5.50-, and 7.70-fold higher than ID8 at the three drug concentrations, P <0.001), while cleavage of caspase-3, lamin A/C, and PARP was attenuated (1.78-, 2.78-, and 1.69-fold reductions relative to ID8 at 125 μmol/L, P <0.001). At 125 μmol/L, MFF upregulation was 1.44-fold weaker (P <0.001), and DRP1 was downregulated yet remained 1.53-fold higher than in ID8 cells (P = 0.015). Consistent with the multidrug-resistant phenotype, ABCB1 was significantly induced, with levels 1.92- (P = 0.023), 2.40- (P <0.001), and 3.80-fold (P <0.001) higher than in ID8 cells at 0, 62.5, and 125 μmol/L, respectively. These coordinated changes suggest that both resistant sublines have undergone adaptive reprogramming of key pathways controlling proliferation, survival, and stress responses, thereby providing the foundation for their acquired drug-resistant phenotype.

## 4 Discussion

In the present study, we observed altered growth patterns and morphology in ID8-T and ID8-C cells compared to parental ID8 cells. This finding is consistent with reports in other drug-resistant cancer models, including carboplatin-resistant retinoblastoma (10) and gemcitabine-resistant intrahepatic cholangiocarcinoma (11), where acquired resistance was accompanied by distinct morphological changes. These observations suggest that morphological remodeling may represent a common adaptive response during the development of chemoresistance, potentially involving cytoskeletal reorganization, altered cell adhesion, or epithelial–mesenchymal transition.

The resistant phenotypes of ID8-T and ID8-C sublines were validated by CCK-8 viability and colony formation assays, both of which demonstrated markedly enhanced drug resistance and clonogenic survival relative to parental ID8 cells. The calculated resistance index (RI) values were 4.54 ± 0.23 for ID8-T and 2.21 ± 0.08 for ID8-C. These moderate RI values are biologically meaningful and reflect the adaptive resistance spectrum commonly encountered in the clinical setting, where dose escalation of first-line chemotherapeutic agents is constrained by systemic toxicity. Consequently, clinical management following acquired resistance typically involves switching to alternative maintenance strategies, such as PARP inhibitors (e.g., olaparib) (2, 12). Consistent with this, our Western blot analysis revealed coordinated alterations in key regulatory proteins in the resistant sublines. Notably, the expression of the growth-related proteins MCM2, STAT3, and 4E-BP1 was modulated. In ID8-T cells, MCM2 expression remained stable under paclitaxel treatment, in contrast to its downregulation in parental ID8 cells—a finding of particular interest given that MCM2 suppression can re-sensitize resistant cells to therapy (13). Similarly, STAT3, a key transcription factor mediating resistance to both paclitaxel and carboplatin in ovarian cancer (OC) (14), showed attenuated downregulation in the resistant sublines. Furthermore, the upregulation of 4E-BP1, which has been linked to ferroptosis and chemoresistance (15), was less pronounced in ID8-T and ID8-C cells. These changes likely support sustained proliferation and stress adaptation, thereby reinforcing the drug-tolerant phenotype.

Paclitaxel and carboplatin primarily exert their antitumor effects through disruption of cell cycle progression and induction of apoptosis (16, 17). Consistent with this mechanism, our flow cytometry analysis revealed that, following drug treatment, both ID8-T and ID8-C sublines exhibited significantly attenuated cell cycle arrest and markedly reduced apoptosis compared to parental ID8 cells. These findings indicate that the acquisition of resistance in these sublines may involve dysregulation of cell cycle checkpoints and/or evasion of apoptotic pathways. Similar observations have been reported in other resistance contexts; for instance, osimertinib-resistant non-small cell lung cancer cells display reduced sensitivity to osimertinib-induced apoptosis and cell cycle arrest (18), underscoring that circumventing drug-induced cytostasis and cell death represents a common adaptive strategy underlying resistant phenotypes. In line with this, our Western blot results showed that, in parental ID8 cells, drug treatment triggered robust apoptosis, evidenced by caspase-3 cleavage and subsequent processing of PARP and Lamin A/C (19). In contrast, ID8-T and ID8-C cells exhibited markedly reduced cleavage of these proteins, indicating a profound blockade of apoptotic execution. Notably, however, under the same conditions, paclitaxel-treated ID8 cells displayed upregulation of cleaved caspase-3 and downregulation of full-length PARP, yet a lack of classical PARP cleavage fragments. A possible explanation is that paclitaxel treatment drives ID8 cells into mitotic catastrophe, in which caspase-3 is partially activated but the apoptotic program is not fully executed (20); concurrently, PARP may be degraded by proteases released from lysosomes (such as cathepsins) at non-canonical sites, leading to reduced full-length PARP in the absence of the classical cleaved bands (21).

Enhanced efflux of chemotherapeutic agents, resulting in reduced intracellular drug accumulation, is a well-established mechanism of acquired resistance (22). Using high-performance liquid chromatography (HPLC) to measure intracellular concentrations of paclitaxel and carboplatin, we found that both ID8-T and ID8-C sublines accumulated significantly lower drug levels than parental ID8 cells, suggesting that upregulated drug efflux contributes to their resistant phenotype. This finding is in line with reports from other cancer types; for example, a similar HPLC-based study in colorectal cancer revealed a 60% reduction in intracellular drug concentration in irinotecan-resistant S1-IR20 cells compared with their parental S1 counterparts (23), highlighting the conserved nature of this resistance mechanism across malignancies. Consistently, our Western blot results demonstrated that ABCB1 (MDR1), an ATP-binding cassette transporter that effluxes chemotherapeutic drugs (24), was markedly upregulated in both resistant sublines. This observation is consistent with the reduced intracellular drug accumulation and represents a fundamental mechanism underlying the multidrug resistance phenotype. Importantly, our cross-resistance assays (**Figure 2**) further support this phenomenon, which is also reminiscent of other established drug-resistant cell lines (25, 26).

Reactive oxygen species (ROS) play a critical role in mediating resistance to paclitaxel and carboplatin in OC (27, 28). To investigate whether similar ROS alterations exist in our models, we assessed intracellular ROS levels following drug exposure. The results showed that ROS levels in the resistant cells were markedly lower than those in their parental counterparts. This observation suggests that modulation of intracellular ROS levels may represent a potential mechanism by which resistant cells escape oxidative stress-induced damage. However, given that oxidative stress constitutes a vast and complex regulatory network, our findings merely provide a preliminary entry point. For example, combined with our Western blot results showing that MFF (which recruits phospho-DRP1 to drive mitochondrial fission and elevate ROS) (29) was downregulated in both resistant sublines, this points to the oxidative stress–mitochondrial axis as a promising direction for further investigation in the context of this cell model.

In summary, although ID8-T and ID8-C were selected by different drugs, they exhibited phenotypic convergence, suggesting a shared multidrug resistance state rather than drug-specific adaptations. These observations outline a convergent adaptive program in which enhanced drug efflux, attenuated apoptosis, and weakened cell cycle arrest act in a coordinated manner. This framework links the seemingly discrete findings of cross-resistance, apoptotic inconsistency, and ROS modulation, and provides a foundation for future functional validation. In the present study, we performed a multidimensional characterization of the drug-resistant phenotypes of ID8-T and ID8-C cells and provided an initial exploration of the biological events potentially contributing to their enhanced resistance. While the mechanistic insights presented herein remain at an exploratory stage and warrant further targeted validation, our findings may serve as a useful reference for subsequent mechanistic studies and highlight several avenues for understanding chemoresistance in OC.

It should be noted that the parental ID8 cell line used in this study is Trp53 wild-type, whereas over 95% of human high-grade serous ovarian carcinomas (HGSOC) harbor TP53 mutations (30). Therefore, while our ID8-T and ID8-C sublines provide a valuable experimental tool for studying chemoresistance mechanisms at the cellular level, they do not fully recapitulate the p53-mutant genetic landscape of the majority of human HGSOC and therefore cannot fully represent human high-grade serous OC in the clinical setting. Consequently, our conclusions regarding the resistance phenotype and associated molecular alterations should be interpreted primarily within the context of p53-wild-type OC models. Nevertheless, the wild-type ID8 model retains its indispensable value in OC research. As one of the most widely used syngeneic models in the field, wild-type ID8 cells provide a defined genetic background and a stable experimental reference, often serving as a critical baseline control for evaluating the relative contributions of drug responses, genetic manipulations, and microenvironmental factors (7, 31, 32). In this context, our well-characterized ID8-T and ID8-C sublines may serve as a valuable reference or comparator for future studies on chemoresistance in p53-deficient OC models.

## Data Availability

The original contributions presented in the study are included in the article/supplementary material. Further inquiries can be directed to the corresponding authors.
